# Occurrence, Persistence, and Virulence Potential of *Listeria ivanovii* in Foods and Food Processing Environments in the Republic of Ireland

**DOI:** 10.1155/2015/350526

**Published:** 2015-10-12

**Authors:** Avelino Alvarez-Ordóñez, Dara Leong, Ciara A. Morgan, Colin Hill, Cormac G. M. Gahan, Kieran Jordan

**Affiliations:** ^1^Teagasc Food Research Centre, Moorepark, Fermoy, County Cork, Ireland; ^2^School of Microbiology, University College Cork, Cork, Ireland

## Abstract

The aim of this study was to assess the occurrence of *L. ivanovii* in foods and food processing environments in Ireland, to track persistence, and to characterize the disease causing potential of the isolated strains. A total of 2,006 samples (432 food samples and 1,574 environmental swabs) were collected between March 2013 and March 2014 from 48 food business operators (FBOs) belonging to different production sectors (dairy, fish, meat, and fresh-cut vegetable). Six of the forty-eight FBOs had samples positive for *L. ivanovii* on at least one sampling occasion. *L. ivanovii* was present in fifteen samples (fourteen environmental samples and one food sample). All but one of those positive samples derived from the dairy sector, where *L. ivanovii* prevalence was 1.7%. Six distinguishable pulsotypes were obtained by PFGE analysis, with one pulsotype being persistent in the environment of a dairy food business. Sequence analysis of the *sigB* gene showed that fourteen isolates belonged to *L. ivanovii* subsp. *londoniensis*, while only one isolate was *L. ivanovii* subsp. *ivanovii*. Cell invasion assays demonstrated that the majority of *L. ivanovii* strains were comparable to *L. monocytogenes* EGDe in their ability to invade CACO-2 epithelial cells whilst four isolates had significantly higher invasion efficiencies.

## 1. Introduction

The genus* Listeria* is at present comprised of fifteen low G+C content Gram-positive species. These are the* Listeria* sensu stricto species* L. monocytogenes*,* L. marthii*,* L. innocua*,* L. welshimeri*,* L. seeligeri*, and* L. ivanovii*, the distantly related species* L. grayi*, and the very recently described species* L. rocourtiae*,* L. fleischmannii*,* L. weihenstephanensis*,* L. floridensis* sp. nov.,* L. aquatica* sp. nov.,* L. cornellensis* sp. nov.,* L. riparia* sp. nov., and* L. grandensis* sp. nov. [1, 2]. Of these, only* L. monocytogenes* and* L. ivanovii* are recognized as pathogenic for warm-blooded hosts. While* L. monocytogenes* causes a severe foodborne disease in humans as well as invasive infections in a range of other mammals,* L. ivanovii* is almost exclusively linked to infections in sheep and cattle, although sporadic cases of* L. ivanovii* associated human infections have been reported [3, 4].

Due to its foodborne transmission, research on* L. monocytogenes* has received special attention in the last decades. Indeed, studies on occurrence and distribution of* L. monocytogenes* in foods and food processing environments are numerous and report variable prevalence. As an example, recent surveys carried out in the United Kingdom [5], Greece [6], Sweden [7], Ireland [8, 9], and various countries in Europe (Austria, Romania, Spain, and the Slovak Republic) [10] have reported* L. monocytogenes* prevalence ranging from 2.5 to 38%. There is less information available in the literature on the occurrence and distribution of other* Listeria* species along the food chain, although it appears that, apart from* L. monocytogenes*,* L. innocua* is the most frequently isolated* Listeria* species [11, 12]. Regarding* L. ivanovii*, a few reports exist which describe a low occurrence, generally of <2% [11–13], although little or no information is available on its occurrence in Irish food industries.

Bacterial persistence, defined as repeated isolation of an indistinguishable (by pulsed field gel electrophoresis [PFGE]) isolate at sampling times greater than 6 months, is a great concern for food industries since it can lead to the repeated contamination of food with spoilage or pathogenic microorganisms and has been demonstrated to recurrently happen for strains of* L. monocytogenes* [14]. A similar phenomenon could also occur for other members of the genus* Listeria*, including* L. ivanovii*. In fact, a study by Vázquez-Villanueva et al. [15] has provided evidence for the persistence of a* L. ivanovii* subsp.* ivanovii* isolate in a Spanish cheese factory. These authors found a common PFGE pulsotype in both ewe's and goat's raw milk batches tested over a 6-month period and on the inner surfaces of raw milk bulk tanks and the milk dump tank at the cheese factory.

Both* L. monocytogenes* and* L. ivanovii* are facultative intracellular bacteria capable of crossing the intestinal barrier and proliferating within macrophages and epithelial and endothelial cells and ultimately inducing cell-to-cell spread [16]. Interestingly, it is well known that* L. monocytogenes* isolates vary considerably with respect to virulence capacity and disease causing potential, with some isolates being incapable of invading gastrointestinal cells due to the expression of a truncated virulence factor, internalin A [17, 18]. Whether similar heterogeneity in disease causing potential is also present in* L. ivanovii* remains unexplored.

The aim of this study was to assess the occurrence of* L. ivanovii* in foods and food processing environments in the Republic of Ireland, to track persistence of the isolates, and to characterize the disease causing potential of the isolated strains.

## 2. Materials and Methods

### 2.1. Detection of* L. ivanovii* in Food and Environmental Samples

From March 2013 to March 2014, a total of 48 food processing facilities from various food sectors, that is, dairy (18 facilities), meat (12 facilities), seafood (8 facilities), fresh-cut vegetable (6 facilities), and miscellaneous (4 facilities), were sampled bimonthly. The selection of food processing facilities allowed coverage of major geographic areas of the Republic of Ireland.

Sampling packs, which consisted of a polystyrene box (DS Smith, UK) containing six premoistened 3M sponge-stick swabs (Technopath, Ireland), a sterile liquid container (VWR, Ireland), two sterile bags (VWR, Ireland), two cable ties, and two ice packs, were sent to all participating food processing facilities. Food business operators (FBOs) received detailed instructions which included information on how to take swab samples, which areas to sample, the type of food samples required, and the packaging and shipment of the samples to the laboratory. For food samples, FBOs were instructed to send two food samples which were at the stage of being ready to be sent from the processing facility.

Every second month, FBOs took 6 environmental samples and sent them to the laboratory by overnight courier along with 2 food samples. Thirty-seven FBOs were initially enrolled in the monitoring programme and 11 further FBOs later showed their interest in joining the collaborative network at different stages during the sampling year. On the other hand, 3 FBOs no longer wished to take part in the analysis or went out of business and several other companies missed one or various sample submissions throughout the sampling period.

Samples were analyzed by following the ISO 11290-1 method for detection of* L. monocytogenes*, except that only one chromogenic agar was used. After the environmental swabs arrived at the laboratory, 100 mL of half Fraser broth (VWR, Ireland) was added to bags containing 3M stick-sponge swabs, after which they were incubated at 30°C for 24 h. Then, a 0.1 mL aliquot was transferred to 10 mL of full Fraser broth, which was further incubated at 37°C for 48 h. In addition, a 0.02 mL aliquot of the 1st enrichment broth was plated onto Agar Listeria according to Ottaviani and Agosti (ALOA) agar plates (Biomérieux, UK), which were incubated at 37°C for 48 h. After incubation of the full Frazer broth, 10 *μ*L was streaked onto ALOA agar plates, which were again incubated at 37°C for 48 h. For liquid or food samples, 225 mL of half Fraser broth was added to 25 mL or 25 g of randomly selected analytical units of the food samples. Samples were then homogenized in a stomacher (Colworth Stomacher 400) for 4 min and incubated at 30°C for 24 h. Subsequently, analysis of samples was continued by following the same approach used for environmental samples. After incubation, ALOA agar plates were examined for typical* L. monocytogenes/L. ivanovii* colonies (blue-green colonies with opaque halo).

After confirmation of* L. monocytogenes/L. ivanovii* isolates (performed as described below) sampling results were regularly communicated to collaborating FBOs.

### 2.2. Molecular Characterization of* L. ivanovii* Isolates

Two characteristic* L. monocytogenes/L. ivanovii* colonies for each positive enrichment were streaked first onto Brilliance Listeria Agar (BLA) plates (Fannin, Ireland), which were incubated at 37°C for 48 h, and then onto Brain Heart Infusion (BHI) agar plates, which were incubated at 37°C for 24 h. Cryoinstant tubes (VWR, Ireland) were prepared by resuspending the bacterial mass from BHI agar plates and were kept at −20°C for bioconservation.

Isolates were differentiated as* L. monocytogenes* or* L. ivanovii* by multiplex PCR as described by Ryu et al. [19] and* L. ivanovii* were confirmed by* sigB* sequencing as described below. PFGE analyses with the restriction enzymes* Asc*I and* Apa*I were carried out on all confirmed* L. ivanovii* isolates according to the International Standard PulseNet protocol [20]. Isolate similarity dendrograms were generated for PFGE analysis using the BioNumerics version 5.10 software (Applied Maths, Belgium), by the unweighted pair group method with arithmetic mean (UPGMA) with tolerance and optimization settings of 1%, as previously described [21]. In addition, representative isolates from each pulsotype were subjected to real-time PCR analyses for differentiation of* L. monocytogenes* and* L. ivanovii* through amplification of* hly* as described by Rodríguez-Lázaro et al. [22] and of* actA* as described by Oravcová et al. [23]. The* sigB* gene of* L. ivanovii* isolates was amplified using Taq DNA polymerase (Thermo Scientific, Ireland) with primers sigB-F (AATATATTAATGAAAAGCAGGTGGAG) and sigB-R (ATAAATTATTTGATTCAACTGCCTT) at 95°C for 2 min, followed by 30 cycles of 95°C for 1 min, 55°C for 30 s, 72°C for 1 min, and a final extension at 72°C for 5 min. PCR products were purified with the QIAquick PCR Purification Kit (Qiagen, Ireland) and sequenced by Source Bioscience services. Phylogenetic relationships between sequences were analysed using the web service http://www.phylogeny.fr/ as described by Dereeper et al. [24].

### 2.3. Invasion of CACO-2 Cells by* L. ivanovii* Isolates

The epithelial cell invasion assay was based upon the protocol of Nightingale et al. [25]. CACO-2 human intestinal cells (originally derived from human colon adenocarcinoma) were routinely maintained and grown in Dulbecco Modified Eagle Medium (DMEM) (Sigma-Aldrich, Ireland), supplemented with 10% Foetal Bovine Serum (Gibco, Ireland), 1% Penicillin-Streptomycin (Sigma-Aldrich), and 1% nonessential amino acids (Sigma-Aldrich) in a 37°C incubator supplemented with 5% CO_2_. Cells were counted using a haemocytometer and trypan blue exclusion to a cell density of 2 × 10^5^ cells/mL of medium and seeded into each well of a 24-well tissue culture plate (Starstedt), in triplicate. Cells were allowed to grow to a confluency of 80% over 48 h. Twenty-four hours prior to the assay, cells were washed and incubated in antibiotic-free DMEM.

Cultures of* L. monocytogenes* EGDe,* L. monocytogenes* PMSC1, or* L. ivanovii* strains were grown overnight in BHI at 37°C with shaking. One mL of the overnight culture was subsequently pelleted by centrifugation and then washed in PBS, diluted to a final concentration of 2 × 10^7^ CFU/mL, and resuspended in antibiotic-free DMEM. Precise numbers of bacterial CFUs added to wells at *T*_0_ were calculated subsequently following plate counts.

Growth medium was removed from the CACO-2 cells in each well and cells were washed once with sterile PBS and 1 mL of bacteria in antibiotic-free DMEM was added (giving a multiplicity of infection of 100). Cells were incubated for 1 h at 37°C/5% CO_2_ to allow for internalisation of the bacteria. Subsequently, the bacterial inoculum was removed and the monolayer was washed once with sterile PBS. Fifty *μ*g/mL gentamicin (Sigma) was resuspended in antibiotic-free DMEM, applied to the monolayer, and incubated for one further hour to kill extracellular bacteria. This was followed by lysis of the entire monolayer with ice cold sterile water containing 0.1% of TritonX-100. One hundred *μ*L of the lysate was serially diluted and plated onto BHI agar (in triplicate for each well) which was incubated at 37°C overnight.

Data were expressed as mean ± SEM of at least three biological replicate samples. Data were transformed to log base ten prior to one-way Analysis of Variance (ANOVA) which was used to test the significance of differences in three or more groups followed by a post hoc test (in this case, Dunnett). In all cases, *P* < 0.05 was considered to be statistically significant. Graphs and statistical calculations were prepared using GraphPad Prism 5 (San Diego, California).

## 3. Results

From March 2013 to March 2014 a total of 2,006 samples (1,574 environmental samples and 432 food samples) were analyzed following the ISO 11290-1 standard methodology.* L. ivanovii* was present in fifteen of the 2,006 samples tested (prevalence of 0.75%), accounting for 14 environmental samples and one food sample. All but one positive environmental sample derived from processing facilities of the dairy sector, where* L. ivanovii* prevalence was 1.7%. These isolates were obtained from nonfood contact surfaces such as drains, floors, and pooled water on floors. The nondairy isolate was obtained from a seafood processing environment (floor), while the positive food sample was obtained from meat sausages. No positive samples were observed in processing facilities of the fresh-cut vegetable sector. It is important to note that only six of the forty-eight processing facilities analyzed had samples positive for* L. ivanovii* on at least one sampling occasion, with prevalence rates at those six facilities ranging from 1.8% to 13.1% (Table 1).

**Figure 1:**
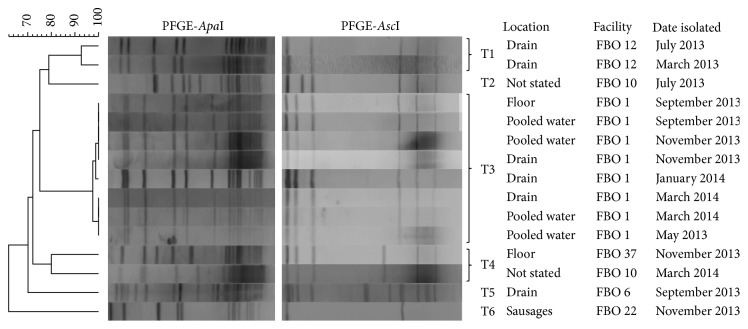
Dendrogram of PFGE pulsotypes of* Listeria ivanovii* isolates obtained from food and processing environment samples from the Republic of Ireland analyzed from March 2013 to March 2014.

PFGE analysis was performed for all confirmed* L. ivanovii* isolates in order to track persistence events in the food processing environment (Figure 1). Six distinguishable pulsotypes were observed. In two dairy processing facilities (FBO 1 and FBO 12),* L. ivanovii* strains with indistinguishable PFGE profiles were isolated at various sampling times during the monitoring programme. For FBO 1,* L. ivanovii* isolates belonging to the same pulsotype were obtained from drains, floors, and pooled water on floors in May 2013, September 2013, November 2013, January 2014, and March 2014 (10-month persistence). For FBO 12, two* L. ivanovii* strains with indistinguishable PFGE profiles were isolated from drains in March 2013 and July 2013.

**Figure 2:**
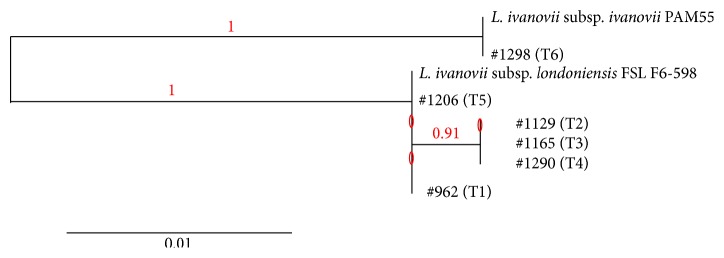
Phylogenetic tree (based on the sequence of the* sigB* gene) for the reference* L. ivanovii* subsp.* ivanovii* and* L. ivanovii* subsp.* londoniensis* strains and representatives of the six* L. ivanovii* pulsotypes found in the current study.

In order to characterize the* L. ivanovii* isolates at the subsp. level, the* sigB* gene was sequenced for representatives of the six distinguishable pulsotypes (Figure 2). Analysis of* sigB* sequences showed that five of the six pulsotypes (which correspond to 14 of the 15 positive samples) belonged to* L. ivanovii* subsp.* londoniensis*, while the remaining pulsotype (T6, with only one strain isolated from meat sausages) was* L. ivanovii* subsp.* ivanovii*.

**Figure 3:**
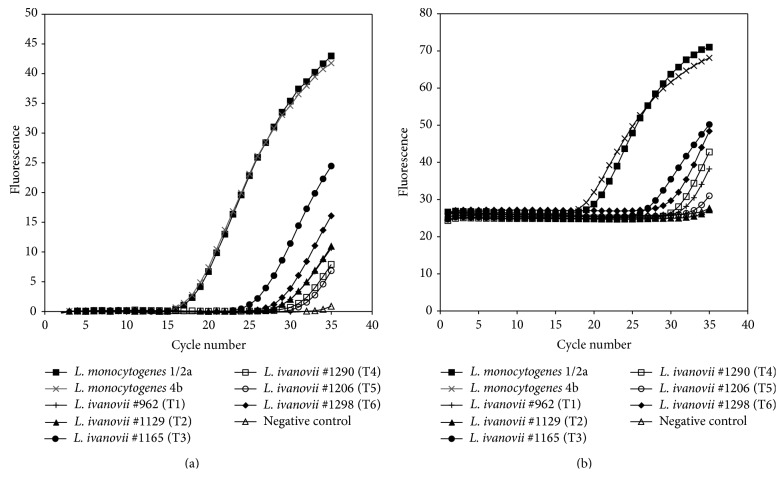
Amplification plot for* hly* (A) and* actA* (B) in* L. ivanovii* following the rt-PCR methodology described by Rodríguez-Lázaro et al. [22] and Oravcová et al. [23], respectively.

When incorporating the PFGE profiles obtained in the current study to the* Listeria* spp. collection of profiles available at Teagasc Food Research Centre Moorepark, it became apparent that several isolates originally confirmed as* L. monocytogenes* by following the real-time PCR approach described by Rodríguez-Lázaro et al. [22] presented PFGE profiles indistinguishable from the ones obtained in this study. Some of these strains were analyzed by multiplex PCR and actually confirmed as* L. ivanovii* (data not shown). Subsequently, the real-time PCR protocol described by Rodríguez-Lázaro and coauthors was applied to representative strains of the six pulsotypes observed in the present study (Figure 3(a)). Amplification of the target* hly* gene occurred for both* L. monocytogenes* positive control strains used, with Ct values of 17.9 and 18.2, while late amplification of the target gene was observed for the* L. ivanovii* isolates tested, with Ct values ranging from 26.1 to 32.7. In addition, the real-time PCR methodology described by Oravcová et al. [23] for confirmation of* L. monocytogenes* based on the amplification of the* actA* gene was also tested with representative strains of the six* L. ivanovii* pulsotypes, and similarly late amplification events occurred, with Ct values ranging from 26.8 to 35.32, in contrast to Ct values of 18.4 and 20.0 observed for* L. monocytogenes* isolates tested (Figure 3(b)).

**Figure 4:**
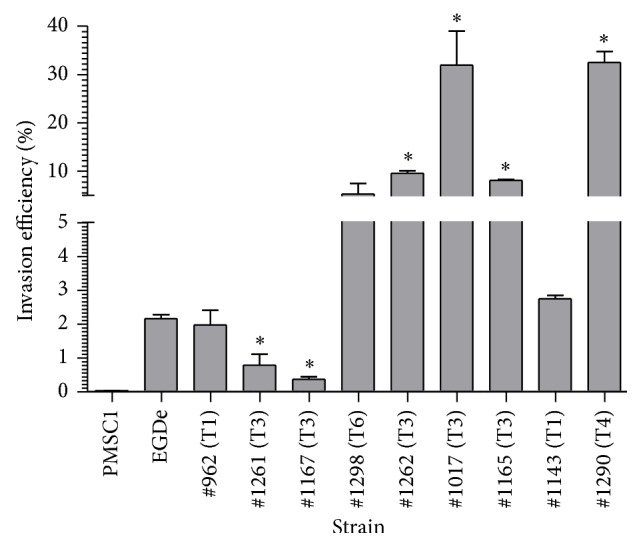
Invasive potential of wild-type* L. ivanovii* isolates in a CACO-2 epithelial cell assay. The strains were incubated with CACO-2 cells* in vitro* for one hour and levels of bacterial invasion were subsequently measured. For comparison, invasive (EGDe) and noninvasive (PMSC1) strains of* L. monocytogenes* were also examined. Data represents % invasion efficacy (relative to* Listeria* numbers initially added per well). Statistical significance was determined using one-way ANOVA and the Dunnett post hoc test with all strains compared to* L. monocytogenes* EGDe (^*^ *P* < 0.05). All strains displayed statistically higher (*P* < 0.05) levels of invasion efficiency relative to the PMSC1 strain.

In order to determine the ability of various* L. ivanovii* strains to invade gastrointestinal epithelial cells, a standardized CACO-2 invasion assay [25] was carried out. Representative strains from 4 of the 6 pulsotypes were compared to an invasive laboratory strain of* L. monocytogenes* (strain EGDe) as well as a noninvasive* L. monocytogenes* strain carrying a defined premature stop codon in the* inlA* gene (PMSC1) [25]. The assay clearly differentiates between invasive and noninvasive* L. monocytogenes* isolates (Figure 4) and invasion efficiency of wild-type* L. monocytogenes* and the PMSC1 strain were roughly equivalent to results in previous studies [25, 26].* L. ivanovii* strains were generally highly invasive with 7 out of 9 strains demonstrating levels of invasion that were equal to or higher than those of* L. monocytogenes* EGDe. Two strains (1261 and 1167) were moderately less invasive than* L. monocytogenes* EGDe, but none of the isolates demonstrated an invasion phenotype that was comparable to the PMSC1* L. monocytogenes* isolate. Interestingly, four* L. ivanovii* isolates (1017, 1165, 1262, and 1290) were significantly (*P* < 0.05) more invasive than* L. monocytogenes* EGDe.

## 4. Discussion

The occurrence of* L. ivanovii* in foods and food processing environments was evaluated for the first time in the Republic of Ireland by bimonthly testing, over a one-year period, samples from forty-eight processing facilities. The observed* L. ivanovii* prevalence was in general low (0.75%). This agrees with the few reports available in the literature which also describe low* L. ivanovii* prevalence in the range 0–2% [11–13]. However, the results showed that* L. ivanovii* occurrence depended on the food sector. Thus, while a higher prevalence of 1.7% was observed for the dairy sector, very low prevalences (0.2% and 0.3%, resp.) were found for the meat and seafood sectors and no positive samples at all were obtained for the fresh-cut vegetable industry sector (278 samples analysed). It is important to note that* L. ivanovii* predominantly infects small ruminants and cattle, which can act as reservoirs. Ruminants can carry* L. ivanovii* and contamination of milk can occur. Interestingly, three of the four dairy business operators that had positive samples (FBO 1, FBO 10, and FBO 12) produce cheese using milk from their own herds of cows or goats. Farming activity is carried out in those cases at facilities close to the cheese making facilities. This may potentially pose a further risk of processing environment contamination by* L. ivanovii*.

A survey regarding* L. monocytogenes* occurrence was conducted in parallel and showed that* L. monocytogenes* was present in 4.6% of samples analysed, with similar rates in food and environmental samples [9]. In most sampling occasions when* L. ivanovii* was detected no* L. monocytogenes* contamination was observed. However, there were three sampling occasions (Facility number 1: Environment, May 13; Facility number 1: Environment, November 13; Facility number 22: Foods, November 13) at which both* L. ivanovii* and* L. monocytogenes* isolates were identified, and in the particular case of Facility no. 1 both* L. ivanovii* and* L. monocytogenes* were isolated from the same samples (a drain and pooled water in the wash room) on November 13.

Molecular analysis of* L. ivanovii* isolates obtained throughout the monitoring programme showed that fourteen of the fifteen isolates (including all dairy isolates) belonged to* L. ivanovii* subsp.* londoniensis*, while only an isolate from meat sausages was* L. ivanovii* subsp.* ivanovii*. Interestingly, all environmental isolates were* L. ivanovii* subsp.* londoniensis*, while the only food isolate was* L. ivanovii* subsp.* ivanovii*. Whether* L. ivanovii* subsp.* londoniensis* is widely more prevalent in the environments than* L. ivanovii* subsp.* ivanovii* or this is a particular phenomenon observed in processing facilities in Ireland remains to be elucidated.

Persistence of* L. ivanovii*, considered for this study as the detection of isolates with indistinguishable PFGE profiles at times six months or more apart, was observed for a cheese processing facility (FBO 1), where a persistent* L. ivanovii* subsp.* londoniensis* pulsotype (T3) was detected repeatedly over a 10-month period (from May 2013 to March 2014) in several nonfood contact environments (drains, floors, and pooled water on floors). In addition, another pulsotype (T1), which cannot yet be considered as persistent, was found in drains of a cheese factory (FBO 12) at times four months apart (March to July 2013). These two cheese processing facilities were the ones with the highest* L. ivanovii* occurrence (13.1% and 4.5%, resp.). Long-term survival of strains in a food processing facility, such as these, confers a higher risk of bacterial transfer to food and therefore a higher risk of human exposure to the microorganism. Bacterial persistence in food processing environments can be due to the existence of harborage sites that are colonized by bacteria and cannot be effectively cleaned or disinfected or can be due to the enhanced ability of some particular strains to grow or survive and therefore persist in industrial settings [27]. Thus, strains with increased resistance to sanitizers, higher adaptability to stress, or better ability to form biofilms might be better suited to persist in inhospitable environments such as those prevailing in food industries. Persistence of* L. ivanovii* in food processing environments has been also previously reported by Vázquez-Villanueva et al. [15] who identified a persistent* L. ivanovii* subsp.* ivanovii* pulsotype from ewe's and goat's raw milk samples from asymptomatic animals at farm level and from swabs obtained from the inner surfaces of raw milk truck tanks and the milk dump tank at the cheese factory level.

The current study also gives evidences that misidentification of* L. ivanovii* isolates as* L. monocytogenes* could occur when following the standard methodology for detection of* L. monocytogenes* in food and environmental samples.* L. ivanovii* strains are phosphatidylinositol-specific phospholipase C positive, and as such they grow in standard selective* L. monocytogenes* chromogenic agar plates forming colonies with the same characteristics as* L. monocytogenes* (blue-green colonies surrounded by an opaque halo on ALOA plates). Genes within the* prfA* virulence gene cluster are habitually used as target genes for* L. monocytogenes* confirmation PCR methodologies (e.g.,* hly* and* actA*). The* prfA* virulence gene cluster is present between the* prs* and* ldh* genes in the pathogenic* L. monocytogenes* and* L. ivanovii* but is absent from the nonpathogenic* Listeria* species [28]. Two widely used rt-PCR methodologies specifically designed for the detection and quantification of* L. monocytogenes* and based on the amplification of the* hly* and* actA* genes [22, 23] were applied to the set of* L. ivanovii* strains isolated in the present study. The results showed that a late amplification (but earlier than the negative control) of both target genes occurred for* L. ivanovii* isolates, which could lead to an erroneous interpretation of results. Indeed, the Teagasc Food Research Centre Moorepark culture collection contained various strains originally classified as* L. monocytogenes* by following the approach described by Rodríguez-Lázaro et al. [22] that were subsequently identified as* L. ivanovii* during the course of this study. These results show the need for fine-tuning of the currently available molecular methodologies for confirmation of* L. monocytogenes*. Incorporation of such molecular tools able to rapidly and successfully discriminate* L. ivanovii* from* L. monocytogenes* is also advisable when implementing monitoring programmes focused on* L. monocytogenes*.

*L. ivanovii* is known to cause disease predominately in ruminants but has been associated on occasions with human disease [3, 4] and is considered to be a potential opportunistic pathogen of humans [4]. To date, studies examining the virulence characteristics of* L. ivanovii* have examined individual reference strains rather than collections of isolates. These studies indicate that* L. ivanovii* is capable of cellular invasion, often at levels in excess of* L. monocytogenes* [29–31].* L. ivanovii* is also capable of lysis of the host cell phagosome and actin polymerization but is perhaps less effective than* L. monocytogenes* in cell-to-cell spread and intracellular multiplication [29, 31, 32]. The findings of this study support previous studies and demonstrated that some wild-type isolates of* L. ivanovii* are more invasive than a clinical* L. monocytogenes* reference isolate (EGDe). Indeed, the majority of isolates in this study were capable of highly effective cellular invasion, suggestive of some degree of disease causing potential. Further analysis is needed to ascertain the precise disease risk associated with these strains but the results suggest that such isolates may pose a health risk for immunocompromised individuals [4].

In conclusion,* L. ivanovii* prevalence in foods and food processing environments in the Republic of Ireland is low but cannot be considered negligible in processing facilities from the dairy sector, where contamination of environments through contaminated raw milk and persistence of isolates with good abilities to grow/survive in industrial settings in particular environments can occur, leading to a higher risk of contamination of processed foods. Although* L. ivanovii* is mainly linked to infections in sheep and cattle, recent reports have highlighted its disease causing potential in humans [3, 4] and the findings of this study demonstrated that the strains described are capable of invasion of human epithelial cells* in vitro*. These findings emphasize the need for dairy processors to be vigilant in order to avoid potential public health risks associated to* L. ivanovii* contamination.

## Figures and Tables

**Table 1:** Occurrence and pulsed field gel electrophoresis characterisation of isolates from *L. ivanovii *positive samples, listed according to processing facility, sampling month, sample type, and pulsotype, for example, T1. Empty white boxes indicate no *L. ivanovii* detected in submitted samples. “—” indicates nonsubmission of samples during that sampling month.

Facility number	% positives	March 13		May 13		July 13		September 13		November 13		January 14		March 14	
		Environment	Foods	Environment	Foods	Environment	Foods	Environment	Foods	Environment	Foods	Environment	Foods	Environment	Foods
Dairy															
1	13.1			**T**3^*^				**T3**		**T3**		**T3**		**T3**	
2	0														
3	0														
4	0														
5	0														
6	2.4							T5				—	—		
7	0	—	—							—	—				
8	0														
9	0														
10	4					T2								T4	
11	0	—	—			—	—	—	—			—	—		
12	4.5	T1				T1		—	—			—	—		
13	0														
14	0														
15	0									—	—	—	—	—	—
16	0														
17	0	—	—	—	—	—	—					—	—		
18	0	—	—	—	—	—	—							—	—

Meat															
19	0	—	—					—	—	—	—	—	—	—	—
20	0														
21	0														
22	1.8										T6				
23	0	—	—	—	—	—	—								
24	0	—	—	—	—	—	—								
25	0	—	—	—	—	—	—			—	—	—	—		
26	0			—	—	—	—	—	—	—	—	—	—	—	—
27	0									—	—	—	—		
28	0									—	—				
29	0			—	—					—	—	—	—		
30	0									—	—				

Seafood															
31	0							—	—						
32	0							—	—						
33	0					—	—	—	—						
34	0														
35	0													—	—
36	0														
37	12.5	—	—	—	—	—	—	—	—	T4		—	—	—	—
38	0	—	—	—	—	—	—								

Vegetable															
39	0														
40	0							—	—	—	—	—	—	—	—
41	0														
42	0					—	—								
43	0														
44	0											—	—		

Miscellaneous															
45	0														
46	0							—	—	—	—	—	—		
47	0	—	—	—	—	—	—								
48	0									—	—				

^*^Pulsotypes which persist in a single facility (isolated at least 6 months apart) are in bold.
